# Women’s experience of intrapartum transfer from a Western Australian birth centre co-located to a tertiary maternity hospital

**DOI:** 10.1186/s12884-016-0817-z

**Published:** 2016-02-08

**Authors:** Lesley Kuliukas, Ravani Duggan, Lucy Lewis, Yvonne Hauck

**Affiliations:** School of Nursing, Midwifery and Paramedicine, Curtin University, GPO Box U1987, Perth, Western Australia; Department of Nursing and Midwifery Education and Research, King Edward Memorial Hospital, PO Box 134, Subiaco, 6904 Western Australia; Family Birth Centre, King Edward Memorial Hospital, PO Box 134, Subiaco, 6904 Western Australia

**Keywords:** Woman-centred, Intrapartum, Transfer, Continuity, Midwife, Birth centre, Labour, Communication

## Abstract

**Background:**

The aim of this Western Australian study was to describe the overall labour and birth experience of women who were transferred during the first and second stages of labour from a low risk woman-centred, midwifery-led birth centre to a co-located tertiary maternity referral hospital.

**Methods:**

Using a descriptive phenomenological design, fifteen women were interviewed up to 8 weeks post birth (July to October, 2013) to explore their experience of the intrapartum transfer. Giorgi’s method of analysis was used.

**Results:**

The following themes and subthemes emerged: 1) The midwife’s voice with subthemes, a) The calming effect and b) Speaking up on my behalf; 2) In the zone with subthemes, a) Hanging in there and b) Post birth rationalizing; 3) Best of both worlds with subthemes a) The feeling of relief on transfer to tertiary birth suite and b) Returning back to the comfort and familiarity of the birth centre; 4) Lost sense of self; and 5) Lost birth dream with subthemes a) Narrowing of options and b) Feeling of panic. Women found the midwife’s voice guided them through the transfer experience and were appreciative of continuity of care. There was a sense of disruption to expectations and disappointment in not achieving the labour and birth they had anticipated. There was however appreciation that the referral facility was nearby and experts were close at hand. The focus of care altered from woman to fetus, making women feel diminished. Women were glad to return to the familiar birth centre after the birth with the opportunity to talk through and fully understand their labour journey which helped them contextualise the transfer as one part of the whole experience.

**Conclusions:**

Findings can inform midwives of the value of a continuity of care model within a birth centre, allowing women both familiarity and peace of mind. Maternity care providers should ensure that the woman remains the focus of care after transfer and understand the significance of effective communication to ensure women are included in all care discussions.

## Background

In Western Australia (WA) 98 % of women birth in hospital [1]. In the 1990s women looking for an alternative option lobbied the government to provide access to a birth centre (BC). Birth centres are small maternity units, staffed by midwives, offering a homely, rather than clinical, environment in order to support women to make informed choices across the childbirth continuum with an aim to birth without medical intervention [2]. The familiarity afforded women with known midwives in a home-like environment prepares them for a labour in which stress hormones are more likely to be reduced so increasing the probability of normal progress of labour [3, 4]. Childbearing women seek out maternity care that is woman-centred and offers informed choice and involvement in decision making [5], indeed women from all walks of life want to have confidence and trust in the staff and simply be treated with kindness [6]. In addition, women opting to birth in a BC are often highly educated and take responsibility for their health, which also includes making informed choices in childbirth planning [7] and they know it is their right to make decisions and take responsibility for them [8]. In 1994 in WA, women looking for such options successfully petitioned for funding which was obtained from the Lotteries Commission, to build a BC alongside the state’s only tertiary referral obstetric unit (OU).

For the majority of women birthing in the BC is straightforward and goes as planned [9], but for some complications occur and it becomes necessary to transfer to the adjacent OU. When this occurs women who are used to making choices and taking responsibility may now be reduced to a more passive role [10], which may affect the woman’s sense of self accountability and control. Alternatively the woman herself may make the decision to transfer, for instance in order to obtain epidural analgesia; this choice may impact on a woman who has prepared herself for natural birth, with no drugs or interventions [11].

Women are affected in a variety of generally negative ways when transfer takes place from a low risk setting to a maternity referral centre [10, 12–14]. In an English qualitative study, 12 women were interviewed who were booked for home or BC birth but were transferred to the local OU in pregnancy or labour [12]. The findings indicated that these women felt a sense of disappointment and failure. Similarly an earlier qualitative English BC study demonstrated a perceived loss of choice, continuity and control which led to feelings of anger and resentment, however as only three of the 18 were transferred during labour, the experiences of intrapartum transfer were not fully explored [14]. In a more recent English qualitative study, 30 women were interviewed who had been booked to BCs either alongside or distant to referral hospitals. In these cases the transfer did take place either intrapartum or immediately after the birth and these women also described a sense of disappointment and a feeling of not belonging during the transfer process [10]. However the timing of interviews was up to one year after the birth which is significant because the length of time between the birth experience and collection of data may impact a woman’s recollections of the labour details so impeding the veracity of the findings.

Findings from this limited number of studies have provided some insight into women’s experiences internationally but in WA the culture of birth represents a different context as a large proportion of women choose to birth with a private obstetrician (41.4 % in 2011 [1]). Recent data indicate that the proportion of births at private hospitals in WA over the past 30 years has increased and now equals the proportion that occurred at public hospitals (42.3 %), excluding the tertiary referral teaching hospital (16.3 %) [1]. Non-hospital births (2.0 %) included mothers who gave birth at a BC (1.2 %) and home (0.8 %). An important influence to this choice is the Australian government’s promotion of private health insurance by imposing a medicare levy surcharge on high income families who do not take out private health insurance [15] which may influence these women to take up the option of private obstetric care. As a consequence of the medicare levy, the rate of people taking out private health insurance has risen and it has been demonstrated that if women have insurance cover they will choose to use it in preference to the public system [16, 17], possibly due to a perception that private health care is the better option. As a result women choosing BC care, which is publically funded, may find themselves swimming against the tide of the opinion of friends and family and so may have more to lose when their plans are undone.

A lack of published literature describing women’s emotions during intrapartum transfer indicated a gap in knowledge, especially because the published literature is from England, where most women birth in the publically funded NHS, compared to WA where 41.4 % of women choose to birth with a private obstetrician. Furthermore the women in the English studies were not only subject to a change in birth environment but also a change in lead professional as the midwife handed over care, which could impact the sense of loss and disappointment the women felt. This study aimed to address the lack of information and awareness in order to help promote a positive labour and birth experience for women when unexpected transfer takes place during this significant life event.

## Methods

The aim of this Western Australian study was to describe the overall labour and birth experience of women who were transferred during the first and second stages of labour from a low risk woman-centred, midwifery-led birth centre to the nearby tertiary maternity referral hospital.

### Design

A descriptive phenomenological study design was chosen in order to capture the lived experience of intrapartum transfer [18] as it facilitates exploring, explaining and describing phenomena in order to interpret their meanings. This method focuses on subjective description in order to gain rich data which provides insight into an understanding of the described experiences [18-21]. The phenomenon in this study was *the overall intrapartum transfer experience* as described by the woman.

### Setting and participants

The study, part of a larger project which also considered the emotions of the partners [22] and midwives [23], was carried out at a BC set in a separate building, alongside the tertiary OU referral centre in WA, with a transfer time from the BC to the OU of 5–7 min. The BC provided woman-centred, midwifery-led care for low risk women in a homelike environment, encouraging family support, partners to stay and use of water during labour and birth. Women were allocated to a group of five midwives during the antenatal period with the expectation that they would meet all midwives in the group during appointments and childbirth education sessions. Women were advised at their first visit that while it was not always certain that they would be familiar with all the midwives in the group, they were reassured that all midwives shared the same philosophy in order to ensure continuity of *care*, if not continuity of *carer*. The shared BC philosophy is based on being woman-centred, facilitating informed choice and helping empower women to help them achieve a vaginal birth with minimal intervention.

In WA from July 2013 to June 2014, 609 women were booked to birth in the BC. Of these 259 (43 %) were transferred antenatally to the OU for reasons such as intrauterine growth restriction or malpresentation. Of the remaining 350 women, 118 (34 %) were transferred in labour leaving 232 (66 %) birthing in the birth centre [9].

During labour woman were encouraged to use non-pharmacological comfort measures, such as double shower and bath but nitrous oxide and oxygen and opiates were available if requested. If an epidural was required or any other intervention for complicated labours, transfer to the co-located OU occurred.

The inclusion criteria for the study consisted of women booked for BC care, who read and spoke English and who were transferred to the tertiary hospital during the first or second stages of labour, accompanied by their partner and the BC midwife. Although the aim of BC care was for all women to be accompanied by a BC midwife when intrapartum transfer took place, this did not always happen. In order to ensure consistency when comparing experiences, only women who were accompanied by a BC midwife were included in the study. Ethical approval was obtained from Curtin University Human Research Ethics Committee (HR91/2013) and the Women and Newborn Health Service (WNHS) Ethics Committee (2013031EW).

### Data collection and analysis

Sampling was purposeful [24] and because each transfer experience is so individual, and therefore impossible to compare like with like, there was no intention to sample women transferred in different situations. Recruitment took place from mid-July to mid-October 2013, with the first author identifying women who met the inclusion criteria from birth records as women who had been transferred during labour, were accompanied by a BC midwife and their partner and who spoke English. The BC midwives caring for the women acted as gatekeepers, by asking them first whether they were prepared to be included in the study. If they agreed the first author approached women prior to discharge from the BC, OU postnatal ward or by telephone within four weeks of the birth. An information letter outlining the aim of the study and consent form were provided at least one week prior to the interview and then signed prior to the interview, if there was agreement to participate. At the beginning of each interview, demographic information such as name, age and educational level was collected from each participant and can be seen in Table 1. Data related to ethnicity, gravidity, parity, length of labour, reason for transfer and type of birth were collected from the woman’s medical record. Women’s ages ranged from 22 to 34, all were Caucasian apart from one woman of Indian origin. Out of the 15 women, 11 were first time mothers with the other 4 having had their second babies. All women apart from two were educated to tertiary level.

**Table 1:** Demographic information

No.		G/P	Education level	Transfer reason	Birth type	BC return post-birth
W1	Peta	1:1	Tertiary	Fetal distress 2^nd^ stage	Vac	Yes
W2	Janine	2:1	Tertiary	Delay 1^st^ stage	SVB	Yes
W3	Susie	2:1	Tertiary	Epidural requested 1^st^ stage	Vac	Yes
W4	Julia	1:1	Tertiary	Epidural requested 1^st^ stage	SVB	Yes
W5	Trudy	2:1	Tertiary	Delay 2^nd^ stage	Vac	Yes
W6	Rosie	2:2	Year 12	Fetal distress 2^nd^ stage	SVB	Yes
W7	Irena	1:1	Tertiary	Fetal distress 1^st^ stage	SVB	Yes
W8	Mandy	3:2	Tertiary	Epidural requested 1^st^ stage	SVB	No (retained placenta)
W9	Judy	1:1	Tertiary	Deep transverse arrest	Em LSCS	No (LSCS)
W11	Deb	2:1	Technical college	Delay 1^st^ stage	Forceps	No (baby to SCN)
W12	Ruth	1:1	Tertiary	Delay 2^nd^ stage	Vac	Yes
W13	Carmel	2:2	Tertiary	Fetal tachycardia	SVB	Yes
W14	Maria	2:2	Tertiary	Undiagnosed breech	Breech vaginal birth	Yes
W15	Diana	1:1	Tertiary	Delay 1^st^ stage	SVB	Yes
W18	Alison	1:1	Tertiary	Delay 2^nd^ stage	Vac	No (3^rd^ degree tear)

*G/P* gravidity/parity, *SVB* spontaneous vertex birth, *Em. LSCS* emergency lower segment caesarean section, *Vac* vacuum/ventouse delivery, *SCN* special care nursery

In order to elicit individual perceptions and meanings, individual in-depth interviews were conducted [18] which began with the broad opening question, “I’d like you to tell me about your birth journey from when the contractions started, to the events leading up to the transfer, your arrival on labour ward up until the birth.”, allowing women the opportunity to give a narrative of their experiences [18]. As the birth story unfolded prompts were used as necessary, to encourage women to describe any part of the experience they felt was relevant, including emotions in the postnatal period based upon reflections of their labour and birth experiences.

The interviews were carried out with 15 women in the naturalistic setting of their homes. They were conducted by the first author, in private in order to minimise any outside influence and all took place within 4 to 8 weeks of the birth, to enhance recall. The interviews were digitally recorded, transcribed verbatim and were read and listened to several more times to ensure accuracy and to maximise immersion in the data. Interviews ranged from 20 to 70 min and a reflexive diary was also completed following each interview to describe overall perceptions and any other relevant information. Data collection and analysis occurred concurrently and recruitment ceased once data saturation occurred, in other words when no new data was being discovered [20].

The transcripts were coded and analysed with the assistance of NVivo. The initial 19 codes [25], were reduced and grouped into themes and subthemes using Giorgi’s descriptive phenomenological method of analysis [22, 26]. Giorgi’s method focuses on descriptions of individual experiences and consists of four steps, starting with immersion in the data by listening and re-listening to the interviews. This is followed by identifying meaning units, which are reduced further by the grouping of similar meaning units leading to an organic formation of meaningful themes and subthemes [25]. Giorgi then suggests putting the focal question to the final themes and subthemes to ensure again that they relate directly to the phenomenon, which in this case was ‘What does this tell me about the woman’s overall experience of *intrapartum transfer*?’ [26]. This process confirmed the validity of the themes and subthemes. To ensure confirmability, once the initial coding had been carried out by the first author, it was also performed independently by the three other members of the research team. Similar themes evolved and all were discussed and corroborated to ensure trustworthiness with the original transcripts. The themes were then synthesised into definitions and linked to direct quotes to illustrate the richness and depth of the participant’s lived experiences.

## Findings

Overall the women felt the midwife’s presence was important to them but while appreciating the fact that help was close at hand, were disappointed to not have achieved the birth they planned for. Five themes with eight corresponding subthemes reflected the variety of experiences and emotions the women felt. These were 1) Listening for the midwife’s voice with subthemes, a) The calming effect and b) Shared philosophy; 2) In the zone with subthemes, a) Hanging in there and b) Post birth rationalizing; 3) Best of both worlds with subthemes, a) The feeling of relief on transfer to tertiary birth suite and b) Returning to the comfort and familiarity of the birth centre; 4) Lost sense of self and 5) Lost birth dream with subthemes, a) Narrowing of options and b) Feeling of panic (see Table 2.).

**Table 2:** Themes and subthemes

Themes	Subthemes
The midwife’s voice	The calming effect
	Speaking up on my behalf
In the zone	Hanging in there
	Post birth rationalizing
Best of both worlds	The feeling of relief on transfer to tertiary birth suite
	Returning back to the comfort and familiarity of the birth centre
Lost sense of self	
Lost birth dream	Narrowing of options
	Feeling of panic

A coding system for each woman was implemented and pseudonyms were assigned and are linked to quotes (noted in italics) to demonstrate confirmability of the findings. Pseudonyms were also assigned to the women’s partners and midwives to ensure confidentiality.

### The midwife’s voice

Women relied on the midwife for advice and were aware that through the labour fog they often focused on the midwife’s voice reminding, advising, informing and generally helping them through. The reassuring guidance was appreciated by Rosie after transfer: *Josie (midwife) was really reassuring and said the birth plan wasn’t completely out the window, we could still have a natural birth.* Reminders of how to breathe through the contractions during the transfer journey were appreciated, for example by Alison: *She kept whispering things in my ear about focusing on breathing and to keep my eyes closed… that really helped,* and Rosie: *She helped me to remember to breathe because there was a time during the labour that I was hyperventilating so she just reminded me to slow my breathing down.* Mandy described the midwife’s voice as a guide which helped her navigate through the events just prior to and during transfer and said her advice to pregnant friends would be to: *Listen to the midwife’s voice, that’s what I remember most when I was in the nightmare of pain and worry; her voice was like a beacon… it gave me a focus.*

#### The calming effect

When navigating the transfer journey and arriving in a new environment women were calmed by the tones of the midwife’s voice, as pointed out by Peta: S*he was very good, calming influence; I remember her calm voice explaining everything.* This was corroborated by Deb: *Like talking me through it, I didn’t know anything that was going on around me but I was hearing nothing but her voice.* This calmness helped many women including Janine who commented that: *Her voice was gentle and encouraging.*

#### Speaking up on my behalf

Many of the women were reassured that all BC midwives shared the same beliefs and philosophy that birth is a normal physiological event. The women felt they knew that the midwives would speak up for them and put forward their views and preferences when necessary, as voiced by Peta: *The midwife had the birth plan with her so I trusted her to have read that and she did…, she knew what we wanted. I felt all the birth centre midwives would know what we wanted.* In the same way Maria stated: *Having that support from a midwife I knew was on the same page in a time of crisis; I knew that her philosophy would be the same as mine.* The value of advocacy was pointed out by Ruth: *Massive. A massive difference because you feel like you’ve got… an advocate there for your well-being and your choices.*

### In the zone

There was haziness and blurring of women’s memories of labour. The women talked of not being aware of time or surroundings and that normal clarity and perception did not exist, as illustrated by Deb: *But it’s just all such a daze… I spent most of the time with my eyes closed, zoned in … time meant nothing, it lasted like forever and it lasted like for no time at all.* The fogginess of trying to recollect the labour was summed up by Irena: *It’s such a blur in my mind* and the lack of clarity was defined by Diana: *There are parts that I don’t remember … because I was zoned out.*

Their labour world was one of being inside themselves, totally withdrawn, almost as if the body was getting on with the job in hand, as remembered by Ruth: *You know I was sort of in that zone of… focus on the labour, like your body’s doing it for you.* Being the woman inside the experience was acknowledged as being different from the observers’ experience, again by Ruth: *Talking to mum and Mike (partner) about it, hearing what they had to go through was, it’s way more scary than actually being the person that it’s happening too; I was in another place in my own head.*

#### Hanging in there

At the point of transfer women knew that they had to try to keep themselves together as Judy described: *I do remember the journey …I was saying can I bring the gas with me? I don’t remember if I was able to bring with me or not now. I just remember was a pretty nasty… it was horrible getting up and getting into the chair.* Trudy acknowledged her readiness to transfer over: *I was getting really really tired and exhausted and just ready to stop the pain,* and Julia*: I didn’t have my eyes open most of the time, I was basically trying to deal with the pain.* Susie knew that transfer was the best option due to her long labour but she couldn’t imagine having to make the journey over:

*I just remember I was in agony, I was trying to get through the contractions when they said they were going to transfer me. I just thought oh can’t they come here? I just felt I couldn’t move, I was in that much pain. I thought I could only be in an upright position and I just thought oh God…I just need to get this baby out, but how on earth am I going to get to the hospital?*

#### Post birth rationalizing

Talking through the birth with the midwife and partner afterwards helped women fill the gaps and revisit what had happened and why. Some women weighed up the decisions made at the time and how they affected the outcome, like Julia*: At the time I was… questioning what would’ve happened if they hadn’t broken my waters and what would’ve been the events after that?* The disappointment Mandy felt following a manual removal of placenta made her question whether it was because she had asked for an epidural which had reduced her mobility: *I don’t know whether if I’d just done it naturally without the epidural whether things would have followed through and I wouldn’t have had a retained placenta.*

Women sometimes spoke of the outcome being the most important result; the fact they ended up with a healthy baby and that the transfer was a very small part of the whole journey, as Ruth explained: *It was just this tiny little bit at the hospital … and then we got to go back… When I look back on the whole experience the hospital bit was the tiniest part of the whole thing.* Some women, like Rosie said they would do the whole thing again and had no regrets: *It was good, I would do it all again.*

### Best of both worlds

Women appreciated the fact that the OU was very close by to the BC. They articulated that they considered themselves to be in the best place if all went well with the familiar home-like environment of the BC but that expert professional help was easily available when necessary, as described by Maria: *I had my ‘homebirth’ but … it was two minutes away from upstairs if anything went wrong.* Janine was thankful that the distance between the BC and OU was relatively short: *I was pleased it was so nearby, no ambulance journey to make.* Similarly Susie felt the same way about help being close at hand: *I was so worried when her heart rate started dropping we needed to get her out, it was great that help was so close by.* In comparison Judy, described how her view shifted, because of requiring an emergency caesarean section, to appreciating the help at hand: *Before I probably had an attitude that … childbirth is totally natural and it’s been medicalised much too much but I ended up falling into the category where I was really glad that there was all of that stuff available to me.*

#### The feeling of relief on transfer to the tertiary birth suite

Women described how the transfer often afforded them a sense of there being light at the end of the tunnel, as Trudy stated: *I was relieved, I felt that at least the end was in sight now* and reiterated by Alison: *It just felt that finally it was going to come to an end and that was a big relief; I just felt like a weight had been lifted off my shoulders.* Added to this was the knowledge that extra support was now at hand, as Janine acknowledged: *I was quite open to some kind of assistance at that point, I was really tired.*

#### Returning to the comfort and familiarity of the birth centre

Women’s experiences in the immediate postnatal period were improved by returning to known midwives in a familiar comfortable environment, as described by Ruth: *I really didn’t want to stay up in the hospital and then Callie (midwife)… said we’ll transfer you in about half an hour and I was so relieved that I didn’t have to stay up there.* Another woman, Carmel was grateful to go back so that her 18 month old first child would be able to join them and spend the night there: *The great benefit of the birthing centre is that whole family can stay overnight.* This was corroborated by Maria who reiterated the value of early family togetherness: *I could have her, Kerry (first child) stay with us, I wanted it to be as normal for her as possible. I didn’t want her to be away from us… the bonding with a new family, that meant so much.*

Similarly Janine talked about the importance of the first night as a new family: *It meant everything… having Harry (partner) stay for that first night together; people who don’t have that really miss out.* The familiarity of the midwives increased satisfaction for Irena: *Knowing the midwives… I mean Poppy (midwife) came in the next day on shift and she came in and saw us… it’s nice to have familiar faces around especially when they’re on your wavelength.*

The experience of returning was particularly beneficial for Susie as it allowed her to emerge from a state of absolute physical and mental exhaustion to a feeling of normality: *Just getting out of there must have helped because as soon as I got to the birthing centre I just felt so much better, like arriving at home, a feeling of peace, comfort, familiarity.* A different perspective was offered by Rosie who interpreted the return to the birth centre as a sign that all must be well: *Coming back was the message to me that everything was good, healthy, normal.*

The women who were unable to return felt saddened by not being able to end the experience in a familiar environment, as expressed by Mandy: *Disappointed. Yeah, because you know down there you can have your family… up there I was by myself the whole time.*

### Lost sense of self

Some of the women found they lost their sense of being included in the events encircling them and instead felt they were being ‘done to’ rather than being ‘part of’. By choosing to birth in the BC women had already made the statement that they wanted to be involved in decision making and make their own choices. However in the transfer process they sometimes felt they were reduced to a non-person because of a different, less woman-centred philosophy, as described by Ruth: *It changed from being caring and focused around what I wanted, to be focused around procedure, without explanation or care or compassion… I felt like I was being treated like a bit of meat rather than a person*. Similarly, a concept referred to frequently by women was the feeling of being left wide-open and vulnerable, as voiced by Alison: *Just not feeling like you have any dignity left … it’s just being naked and exposed* and Mandy: *You felt really undignified…they strap your legs upon stirrups, you don’t really get told that’s what’s going to happen.*

In a similar way women discovered that in this new position they were diminished and became a teaching tool for junior doctors and students and in some cases found they were being observed by many maternity care professionals because they were an ‘interesting case’. An example of this is Maria, who arrived in the BC with a breech presentation and described how, after transfer to the OU, she had to close her eyes for the birth in order to try to recapture a sense of being a woman birthing a baby: *There were too many… lots of people and that freaked me out so I just didn’t want to have to look at them*.

### Lost birth dream

Women voiced disappointment that their planned birth was never achieved. Throughout the pregnancy and up until the point of transfer they had visualised a calm and peaceful birth with personal choices such as water birth, the cord to be left pulsating, the baby to be skin to skin, but the eventual reality was that for many the opposite happened. The sadness of not achieving the anticipated plan was considered by Diana: *Because I’d always wanted a water birth that’s why we went with the birthing centre… so I didn’t get to have what I wanted… I was disappointed.* In comparison some of the women were saddened but accepted the transfer as necessary, for example Trudy: *I was disappointed but at the same time knew that we had to do what we had to do so* and Rosie: *A bit upset because I really wanted the water birth but at the same time it was okay, we were doing what we needed to do.*

Interestingly some women felt the disappointment happened because they perceived that they had set themselves up for failure by preparing for the perfect birth and presuming that everything would go according to plan, as Ruth vocalised: *But it’s so true because the higher you set your goals the more disappointed you will be if you don’t get there… I set myself up; I set my goals too high.*

#### Narrowing of options

After transfer took place the realisation dawned that care was managed with a different focus. There was a sense of urgency and also an expectation that the women would conform; they would lie on the bed, they would be continuously monitored with a cardiotocograph (CTG) machine, they would follow directions and accept the decisions made about their care, as Trudy remembered: *Pretty much all my birth preferences went out of the window. Things like waiting for the cord to stop pulsating and that sort of stuff.* In a similar way Ruth found it hard to have to relinquish the bath: *I just wanted to get back in the bath and she was like no… I’m sorry but you can’t get back into that bath.* The discomfort of continual fetal monitoring and the immobility it caused was voiced by Maria:

*In a hospital, you can’t do any of the things that make you comfortable; I couldn’t move around like in the birth centre, and I had this heart rate monitor which is a big plastic thing and every time I bent over I pushed it off and they had to keep putting it back on… that really affected my experience because I had to worry about how I was standing to make sure the monitor kept working.*

#### Feeling of panic

The transfer brought with it a sense of urgency which many women likened to being in an episode of a hospital emergency drama program. The feeling of being rushed and losing her partner’s hand was described by Peta: *I was holding on to Robbie’s (partner) hand and I wasn’t allowed to do that while I was on the trolley because there wasn’t enough room and so I was gripping onto the side and they kept saying stop gripping onto the side.* Being raced up to the OU was outlined by Julia: *I could tell that they were racing up with the bed bumping into things.* Similarly the dramatic change from calm to drama was summed up by Alison: I*t was like lights, camera, action*.

## Discussion

The main findings from this qualitative study demonstrate that when women were transferred in labour, they were affected by disruption to their prior expectations and they felt that various factors helped or hindered the process. The alteration to their labour journey was eased by the BC midwife’s presence, providing continuity of midwifery care and a calming influence. When the time came to transfer many felt relieved that help was close at hand but despite this they often felt vulnerable and exposed after arrival in the tertiary birth suite and were aware of a change in attitude and behaviours towards them. There was a sense of disappointment at leaving the familiarity of the BC and losing their planned birth and women were appreciative when they were able to return to the BC again afterwards. The labour phenomenon of women ‘being in the zone’ was confirmed in this study with women wanting to talk about the journey afterwards to be able to fill in the gaps and rationalize what had happened.

The value of the phenomenological approach of the study was used to give a window of insight to allow maternity providers to appreciate the woman’s lived experience [18]. This method allowed depth and richness of description from the women during a time that was close enough to the birth to enable recollection of their experiences. The interviews were not time-limited which gave women the freedom to carefully explore their labour memories. These methods provided a wealth of information which is not normally shared and can give an enhanced understanding of women’s experiences of intrapartum transfer. Interviews from the women in this study demonstrated that they valued being accompanied by a BC midwife when transferred to the OU and appreciated knowing that they shared the same beliefs and philosophy. Women also commented on hearing the midwife’s voice through the labour haze and the fact it was calming and reassuring when they needed it most. Midwives’ sensitivity to women’s cues in labour regarding the nuances of communication and remaining calm and connected, to enhance the labour experience [22, 27, 28], was also confirmed in this study. The woman-midwife relationship is multi-faceted and trust and mutuality are enhanced with insight and awareness from and the presence of the midwife [29].

Findings from previous studies [10, 12], suggesting that transfer in labour causes disappointment to women, were endorsed in this study which clarified that these feelings were due to prior expectations being disrupted and choices reduced. Constraints such as such as being confined to bed, unable to use the bath or shower and needing to conform to such practices as continual fetal monitoring made women feel restricted and uncomfortable, which is also described in previous studies [30, 31]. For some women the transfer brought a feeling of relief [10], often underlined by the knowledge that the OU was close by. Women were reassured that the BC setting offered the best of both worlds with the advantage of a home-like environment and woman-focused care but the back-up of medical expertise just minutes away if necessary [10]. The opportunity to return to the BC afterwards closed the circle for many women and gave a feeling of relief and returning home.

On arrival at the OU many women were concerned about the change in attitude and behaviours towards them. Women in labour are very vulnerable [32] and need close attention and care but often the focus of maternity care staff is on the fetus rather than the women herself [33]. This study revealed that women felt the anguish and humility of being a vessel of the fetus rather than an individual person. Similarly women spoke of feeling exposed and vulnerable and experienced a loss of dignity from being used as a teaching tool for the benefit of staff. Although many maternity facilities are also teaching hospitals to benefit future generations of obstetricians and midwives, the privilege of being able to learn a skill on another human being should never be taken for granted and the vulnerability of the woman must be carefully considered. Feelings of having been violated and the subsequent threat of dealing with post-traumatic stress disorder are well recognised in women who suffer trauma during labour and birth [34, 35]. These known anxieties, confirmed by the tearful episodes demonstrated by several women during interviews in this study provides maternity care providers with the evidence that the woman’s birth space is where her wishes should be supported, birth attendants limited and dignity preserved.

The instinctive behaviour of women in labour was highlighted in this study and how it impacts on their experiences during labour and also their memories of labour in the few weeks afterwards. Getting into the zone is a well-known phenomenon [36, 37] but this study also demonstrated how after the birth women became aware of the need to fit missing pieces of the jigsaw and fill gaps in their memory. This WA study confirms the value of revisiting the birth through a conversation afterwards [38] but also that it should always take place with reference to the woman’s medical record in order to enlighten the woman accurately and ideally with the midwife who looked after her in labour.

The limitations of this study include the fact that women were only eligible to take part in this study if they were accompanied during transfer by their partner and a midwife. This excluded valid experiences of women who were transferred without the benefit of an accompanying BC midwife and the difference this might have made. Similarly there was no maximum variation sampling used, so the circumstances of transfer, for example, whether the transfer was for a ‘non-urgent’ reason or an emergency was not taken into account and this could also be an important factor in the transfer experience. Another limitation is that it is a small study that only reflects the experiences of women in one Australian birthing centre however a rich description has been provided to allow the reader to determine the transferability of the findings to other contexts.

## Conclusions

Women in WA felt that on intrapartum transfer from their chosen birth centre to a tertiary obstetric unit, they lost the birth dream they had been visualising and planning for. It has been established that ongoing conversations between the woman and midwife during the antenatal period increases preparedness and facilitates a more satisfying labour experience [39] and could also prepare women for an altered labour journey. Continuity from the midwife plays an important role in preparation and also in helping the woman transition to a new environment if intrapartum transfer becomes necessary. The current emergence of midwifery group practices in Australia following the recommendation of the National Maternity Services Plan [40] will allow more Australian women in the future access to this preference.

The Western Australian Reid report [41] recommends that more women should have access to birth centres as they are known to improve maternal satisfaction across the childbirth continuum [42, 43]. Women’s appreciation of BC care was corroborated with this WA study in which they voiced that choosing to birth in an BC co-located to an OU offered the best of both worlds. They felt reassured that extra help was there if necessary and often relieved when the transfer took place, but were very pleased to be able to labour as far as possible in the BC and then return to its familiarity afterwards. This recommendation should be considered by policy makers in the health sector when the value of birth centres is being questioned; current birth centres should not be threatened with closure and new birth centres considered for any new or existing maternity facility.

Information that has emerged from this study, that women are committed to try to achieve aspects of their birth plan even after transfer to the OU takes place. Midwives may now be encouraged to consider simple measures such as mobilisation and use of water in OUs, despite the woman being subject to intervention, which will also reduce length of labour, reported pain and increase satisfaction [44].

To increase inclusion for women in decision making, health care professionals should ensure that sensitive communication channels are kept open at all times so that women can continue to make choices and feel involved in their care. The need for true informed consent has been well documented in the literature [45, 46], but is still not universally offered. These corroborated findings direct maternity care providers to ensure this is obtained in all cases. The experience of being excluded was described by women as having left their sense of ‘self’ back in the birth centre and their dignity and modesty were compromised as they were treated as a vessel for the baby rather than as a person in their own right. Maternity care providers should ensure that women are treated with dignity and respect and remain central to care, rather than focussing solely on the wellbeing of the fetus. These findings direct maternity care providers to widen their focus from fetal wellbeing to considering the woman as her own person.

The midwife’s voice is a point of reference for women during labour and can be used to help focus the woman, impart important information and maintain a sense of calm and normality even when problems occur. It is well documented that there is always room for improvement with communication throughout labour [47, 48] but this new knowledge will give midwives impetus to inform, advise and reassure women when required. After the birth women were aware that they had unclear memories that midwives could help clarify by offering time to talk through events to fill in the missing pieces of the jigsaw.
